# Lithium chloride treatments in free flying honey bee colonies: efficacy, brood survival, and within-colony distribution

**DOI:** 10.1007/s00436-023-08084-y

**Published:** 2023-12-22

**Authors:** Carolin Rein, Marius Blumenschein, Kirsten Traynor, Peter Rosenkranz

**Affiliations:** State Institute of Bee Research, University of Hohenheim, 70599 Stuttgart, Germany

**Keywords:** *Varroa destructor*, *Apis mellifera*, Lithium chloride, Brood survival, Distribution

## Abstract

The efficacy of various lithium chloride (LiCl) applications in eradicating the parasitic mite *Varroa destructor* in honey bee colonies was investigated, with a specific focus on its impact on brood development. In broodless colonies (3 weeks post queen caging), the highest efficacy of 98% was achieved with a 9-day treatment of 2.5 kg of candy spiked with 50 mM LiCl. A shorter 5-day treatment with 2 kg of 50 mM LiCl candy resulted in an efficacy of 78%. In colonies with brood, a repeated short-term application of 4 × 0.5 kg 50 mM LiCl candy yielded an efficacy of 88%. LiCl treatment led to a removal of the first batch of brood reared after release of the queen. However, no long-term effects on colony growth were observed, and the colonies successfully overwintered. Additionally, the study demonstrated that lithium is rapidly distributed among the bees of a colony within 2 days, yet only low concentrations were detected in stored food samples. This suggests that the bees efficiently absorb and distribute lithium within the colony. The harvested honey in the following spring revealed a lithium concentration of 0.1–0.2 mg/kg, which is below naturally occurring lithium levels in honey. Based on these findings, LiCl can be considered an effective and easy-to-apply acaricide in broodless colonies, and even in colonies with brood, it had good efficacy and no long-term effects on colony survival. Further research may be necessary to determine the optimal treatment period for achieving an efficacy over 95%.

**Supplementary Information:**

The online version contains supplementary material available at 10.1007/s00436-023-08084-y.

## Introduction

The negative impacts from globalization are exemplified by the worldwide spread of the honey bee mite *Varroa destructor* (Anderson and Trueman 2000), a striking example of the redistribution of diseases and parasites in wildlife and livestock (Travis et al. 2011). *V. destructor* is originally a parasite of the Eastern honey bee *Apis cerana* (Fabricius 1793) in Asia. Intensive trade of managed *Apis mellifera* (Linnaeus 1758) colonies during the last century enabled the mite’s shift to this new host and promoted worldwide distribution (Chantawannakul et al. 2016; Chapman et al. 2023).

*V. destructor* causes severe morbidity and mortality of entire *A. mellifera* colonies by feeding on host tissues and transmitting viruses (Rosenkranz et al. 2010; Traynor et al. 2020). Regular Varroa treatments are indispensable because without beekeeper intervention, colonies dwindle (Genersch et al. 2010; Gray et al. 2019; Hernandez et al. 2022; Seitz et al. 2015; Stahlmann-Brown et al. 2022). Colony losses due to Varroa infestation continue to be high for two main reasons: firstly, there has been limited success in breeding viable Varroa-resistant honey bees (Büchler et al. 2010; Mondet et al. 2020a, b) and secondly, the biology of *V. destructor* with the reproductive phase inside the protected brood cell hinders the effectiveness of treatments (Traynor et al. 2020). When brood is present in the colonies, reproductive mites are located within the capped brood cells (Fuchs 1985, 1992; Ifantids 1988) and thus protected against nearly all common acaricides, except formic acid (Rosenkranz et al. 2010). A further challenge to controlling *V. destructor* under temperate climate conditions is that (i) chemical treatments should be avoided during nectar flows when bees collect nectar to make honey yet (ii) a treatment must be performed in late summer before the bees rear their long-lived winter bees, so that their longevity is not compromised through high levels of parasitization. High mite infestation levels in late summer and the subsequent damage to winter bees, as mite levels continue to be high in fall, are the main drivers of winter colony losses (Genersch et al. 2010; Gray et al. 2019; Seitz et al. 2015). The relatively small window of time for late summer treatment increases the need for highly effective varroacides in mite management strategies. Several varroacides are registered; however, the majority are based on only a few active compounds such as formic and oxalic acid, thymol and a few synthetic substances from the group of pyrethroids, formamidine, and organophosphate (Mutinelli 2016; Qadir et al. 2021; Vilarem et al. 2021). None of these registered products fulfill all desired characteristics of an “ideal” veterinary drug: (1) high and reliable efficacy, (2) limited side effects, (3) no residues in bee products above critical thresholds, (4) low risk of mite resistance, and (5) easy-to-apply. The widely used organic acids are poorly tolerated by bees or brood with significant impacts on colony size, especially in warm climates (Bubnič et al. 2021; Elzen et al. 2004; Rademacher et al. 2017; Satta et al. 2005). In particular, formic acid, a common agent used in late summer as it penetrates into the brood nest, requires specific environmental conditions and its efficacy is strongly influenced by colony factors, such as colony size and amount of colony thermoregulation (Pietropaoli and Formato 2019; Steube et al. 2021; Underwood and Currie 2003). Repeated applications are necessary to achieve sufficient efficacy, which makes the use of organic acids time-consuming and thus costly, especially in large beekeeping operations (Berry et al. 2022). Synthetic varroacides often result in accumulating levels of residues in beeswax, with potential impacts on later larval survival and queen quality (Albero et al. 2023; Haarmann et al. 2002; Kast et al. 2021), and frequent reuse leads to the development of mite resistance (Higes et al. 2020; Mitton et al. 2022). Due to these shortcomings of the current options for chemical control of Varroa, there is still urgent demand for further varroacidal compounds.

The recently discovered lithium chloride (LiCl) meets many of the above mentioned requirements of a varroacide (Ziegelmann et al. 2018), and so we wanted to examine its efficacy and distribution in free flying colonies. LiCl is a widely distributed salt (Szklarska and Rzymski 2019), a natural component of honey (Abdulkhaliq and Swaileh 2017; Bogdanov et al. 2008; Conti et al. 2018; Karabagias et al. 2017; Tariba Lovaković et al. 2018), and is used in therapeutic treatments of bipolar disorders and depression (Ferensztajn-Rochowiak et al. 2021; Gomes-da-Costa et al. 2022). LiCl is very effective at killing mites via contact and displays a systemic mode of action, while being well-tolerated by adult honey bees (Kolics et al. 2020, 2021b; Ziegelmann et al. 2018). A limitation for a broad applicability of LiCl is currently the low tolerability by honey bee brood (Rein et al. 2022).

Climate change alters the way beekeepers manage colonies, as often colonies no longer have a natural brood break which enables an effective winter treatment. In southern Europe, it has therefore become common to cage queens after the summer honey harvest and then treat the mites in the dispersal phase on adult bees when colonies are broodless (Büchler et al. 2020; Lodesani et al. 2014). LiCl is an ideal compound under these circumstances, due to its high efficacy and good tolerability by adult bees (Stanimirovic et al. 2022; Ziegelmann et al. 2018). Some field tests with lithium salts have already been performed; however, most of them used a repeated trickling method similar to the application of oxalic acid (Jovanovic et al. 2022; Kolics et al. 2021b, 2022).

In our approach, we wanted to capitalize on the systemic mode of action of LiCl, i.e. the administration of the active substance via food to the colonies. This new application method would enable a quick and “easy-to-apply” treatment. We present the efficacy and distribution of LiCl using different food applications in broodless colonies under realistic field conditions with a special focus on the subsequent development of the brood. In the trial of 2022, we compared the broodless application with repeated short-term treatments in brood rearing colonies. We hypothesized that such short-term treatments would reduce the exposure time and risk of honey bee larvae to LiCl and therefore potentially reduce the loss of brood as described in Rein et al. (2022).

## Materials and methods

### Experimental setup

Field trials were performed in two apiaries. The preliminary experiment in 2018 took place at the field station Heidfeldhof of the University of Hohenheim (48°42′55.3″N 9°10′49.2″E), whereas the main experiments in 2021 and 2022 were performed in the local apiary of the State Institute of Bee Research at the University of Hohenheim (48°42′32.7″N 9°12′38.9″E). Each colony consisted of a total of 20 frames in two Zander size brood boxes with similar colony strength—estimated by the number of combs covered with bees and the number of brood combs—and headed by healthy sister queens. The queens originated from the local “wildtype” stock managed by the State Institute of Bee Research, University of Hohenheim, Stuttgart, Germany.

**Fig. 1:**
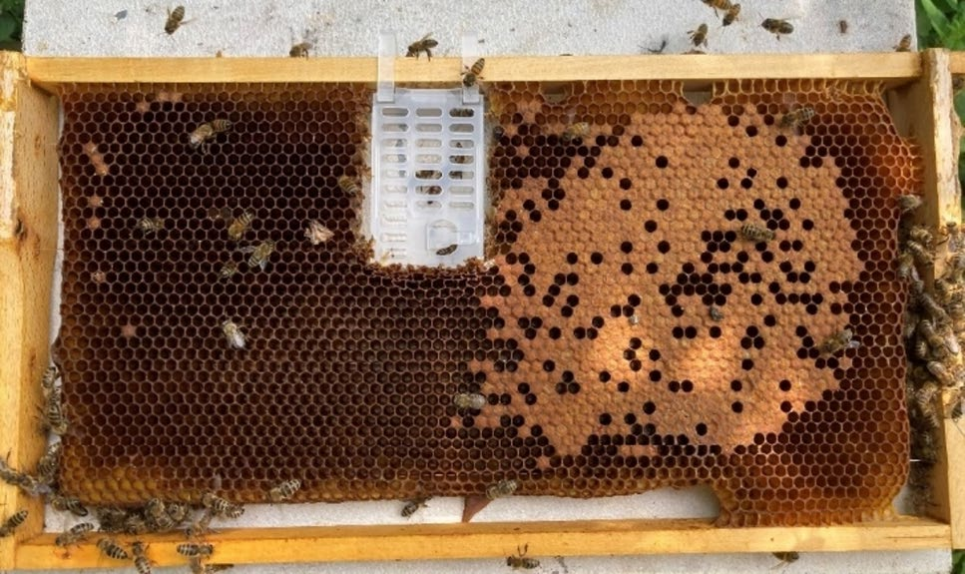
Queen is caged inside the Rubee® Varroa control box inserted into a brood frame, which prevents her from laying eggs and allows the colony to achieve a broodless phase

The experimental setups of the different trials are shown in Table 1. Our main objective was to investigate different applications of LiCl in broodless colonies during summer, so to achieve broodless colonies we first had to cage the queen. After the last honey harvest in summer, the queen of the respective colony was placed in a small plastic cage (Varroa control box, Rubee®) on a frame in the middle of the brood nest in the bottom hive body at the start of each trial (Fig. 1). The cages are designed with queen-excluder sized material, allowing worker bees to pass through while confining the queen. After three weeks, when the last of the brood emerged, the queen of the respective colony was released and the treatment with LiCl started on the same day.

**Table 1:** Specifications of the tested treatment methods with acronym, LiCl concentration used, treatment period, number of tested colonies, and intervals of data collection for the preliminary and main experiments

Treatment method		LiCl concentration	Treatment period [days]	No. of colonies	Start of brood assessment	Sampling interval of bees and food
Preliminary experiment						
Positive control:	250 ml formic acid; Nassenheider®	–	8	4	–	–
P1:	7 l syrup; 1 day after ROQ*	25 mM	8	4	–	–
P2:	4.5 kg candy; 1 day after ROQ*	50 mM	8	5	–	–
Experiment I, 2021						
Positive control:	250 ml formic acid; Nassenheider®	-	9	9	–	–
A1:	2.5 kg candy; on the day of ROQ*	50 mM	9	6	5 days after application	4 days
Experiment II, 2022						
A2:	2 kg candy; on the day of ROQ*	50 mM	5	10	3 + 19 days after application	2 days
A3:	4 × 0.5 kg candy in 7 day intervals; breeding colonies	50 mM	4 × 2	10	0 + 16 days after 1st application	2 days

*ROQ release of queen

In the preliminary experiment, we tested two different LiCl applications with 25 mM LiCl syrup and 50 mM LiCl candy and compared the efficacy to positive control colonies treated with 250 ml formic acid (60%, Nassenheider® evaporator) (Table 1). The concentrations were chosen based on previous experiments by Ziegelmann et al. (2018) with artificial swarms.

**Fig. 2:**
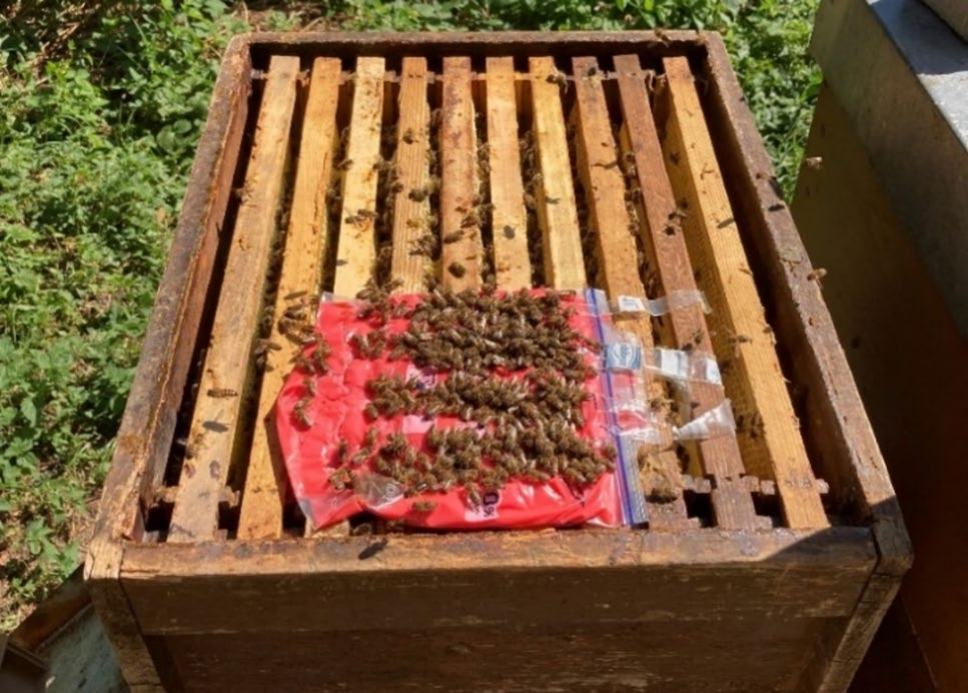
Treatment application (A2) of 50 mM colored LiCl candy in plastic bags on top bars of frames. Slits were cut into the bags to allow access to the bees

In the main experiments, we used 50 mM LiCl candy, as it was the more promising approach due to ease of application combined with a slower, more consistent consumption of the LiCl food. In 2021, we tested a single application (A1) of 2.5 kg 50 mM LiCl candy (fed over a period of 9 days via a plastic bowl protected by an empty honey chamber) and compared it with the positive control group, treated with 250 ml formic acid (60%, Nassenheider® evaporator) (Table 1). In 2022, we modified the LiCl application and fed 2 kg 50 mM LiCl candy via small Ziplock plastic bags (1 l SafeLoc®, Toppits®) put directly on top of the frames (A2) (Fig. 2), due to ease of application and to provide ready access for the bees. We also shortened the treatment period from 9 to 5 days to eliminate exposure of the newly developed larvae with LiCl. A flipped top feeder (Nicot®) provided enough space for the bees to access the treatment food.

To test whether a repeated short-term application (A3) can prevent brood removal with the same Varroa control efficacy, we applied 0.5 kg 50 mM LiCl candy four times in 7-day intervals (in total 2 kg candy) without caging the queen, meaning that all brood stages were present during the treatment. The plastic bags containing 0.5 kg candy were emptied within 2 days of application, therefore the treatment period was defined as 4 × 2 days. Untreated colonies were not used as a negative control to avoid mite reinfestation by highly infested nearby colonies (Frey and Rosenkranz 2014), which would result in inaccurate calculations of efficacy.

Data were collected on Varroa mite mortality, brood development, and distribution of lithium in sampled bees and stored food as described below.

### LiCl food production

For the syrup, we used Apiinvert® (saccharose, fructose and glucose, Suedzucker Group^©^) and mixed it with the respective amount of lithium chloride (> 99.9%, p.a., ultra-quality, Roth®) to reach a 25 mM concentration (1.06 g LiCl-salt per liter). We added food coloring (“Tannengrün”, Städter®) to the syrup, which dyed it green, so we could identify where the syrup was stored in the combs. Such food coloring has no negative impact on honey bees or honey bee brood (Ehrenberg et al. 2019).

The candy was made from powdered sugar (Suedzucker Group^©^) and honey harvested during summer nectar flows, mixed in the ratio of 2:1. Additionally, a concentration of 50 mM LiCl and red food coloring (Allura Red AC, Sigma-Aldrich®) was added into the mixture. To reach this concentration, we used 1.57 g LiCl-salt dissolved in water for 1 kg of candy. This was mixed until the food coloring was evenly distributed using a commercial dough mixer (ITR50 2 V “Evo”, Prismafood DE). To determine the exact amount of LiCl used per colony, food consumption was measured by weighing the remains of the applied food upon removal.

### Varroa mite mortality and calculation of efficacy

The mite mortality was recorded by counting the mites every two days on the sticky board inserted under the screened bottom board of the hive, which was not accessible to bees. The sticky board is a tray prepared with a single layer of kitchen paper towel, moistened with oil, which prevents other insects from removing the dead mites and which was renewed after each mite mortality count. The natural mite drop was recorded two weeks prior to the treatments. Dead mites found on the sticky board during LiCl food application and the consecutive 7 days were considered killed by LiCl. For the positive control colonies treated with formic acid, we considered dead mites on the sticky board from 1st to 20th day after treatment administration. Formic acid penetrates the sealed brood cells to kill mites inside capped cells, which is why we extended the monitoring period to cover an entire brood cycle.

Each colony received a follow-up treatment for four to six weeks with either Bayvarol® (2018 and 2022) or Apivar® (2021). We alternated the compound for the follow-up treatments every year to avoid development of mite resistance. In addition, the good efficacy of both, Bayvarol® and Apivar® has been confirmed by field trials conducted on the Hohenheim campus in the previous years.

The efficacies of the treatments were calculated on the basis of mite mortality in the test colonies. The following formula was used according to standard guidelines for control of *V. destructor* (Dietemann et al. 2013; EMA 2021; Pietropaoli and Formato 2019; Semkiw et al. 2013), where the number of mites killed by the treatment is divided by the total number of mites that fell including those killed by the follow-up treatment:

$$\%\;efficacy=\frac{no.\;of\;mites\;killed\;by\;treatment\;\times\;100}{no.\;of\;mites\;killed\;by\;treatment\;+\;no.\;of\;mites\;killed\;by\;follow\;up\;treatment}$$

### Honey bee brood survival

To evaluate the effects of the different LiCl treatments on brood, we conducted a brood assessment of newly laid eggs in the main experiment. We marked the position of eggs on a transparent acetate sheet (= brood area fixing day (BFD)), according to the method described by Schur et al (2003). We inspected the viability of the brood every four days and marked cells with viable larvae as well as cleared out cells on a new transparent sheet. As eggs do not hatch for three days, the exact age of the larvae varied between 1 and 72 h. To keep the error rate low and to guarantee that empty cells were cleared out and the bees had not emerged, we terminated the assessment on day 16 (BFD + 16). From these data, we calculated the brood survival rate for each assessment.

After being caged for three weeks, the queen typically requires a few days to start laying eggs again, therefore we started the brood assessment in treatment group A1 five days after the release of the queen and the start of the treatment application. In treatment group A2, we were already able to find enough eggs three days after queen release. In 2022, we not only examined the development of the first brood cycle, but also a second brood cycle 16 days later to evaluate possible long-term effects (Table 1) for both the A2 and A3 treatment groups.

### Distribution of lithium among worker bees, stored food, and residue in honey

An even distribution of the active component lithium among the worker bees is important for a sufficient treatment efficacy. In contrast, the chloride anion is not significant (Ziegelmann et al. 2018). Thus, we utilized ICP-MS analysis to determine the concentration of lithium in the samples. In 2021, we took bee samples (approx. 30 bees) from each treated colony at regular intervals during and post treatment (as shown in Online Resource 1). In 2022, we took bee samples from the central frames of the hives every other day for three weeks in trial A2 and for four weeks in trial A3, as well as collecting additional bees 39 days post-treatment from both groups. Stored food samples were taken once a week until day 39 post-treatment from four combs containing food stores in the top hive body.

To investigate whether the LiCl treatments had an effect on the lithium levels in food and honey of the following year, we took a sample from the brood chamber of each colony before installing the honey chamber on 19th of April 2023. Three weeks later, we took samples from the honey chamber of each colony that already collected nectar. On the 9th of June, the honey was harvested for each treatment group individually (A2 and A3) and samples were analyzed.

After sampling, the bees were frozen (− 20 °C) until dissection of the honey crop. Bees were thawed, and we gently pulled on the abdomen with forceps, which exposes the crop. The crops of 20 bees per sample were pooled, homogenized, and analyzed with ICP-MS (Inductively Coupled Mass Spectrometry) for the concentration of lithium. The samples of stored food from the colony were also frozen (− 20 °C) until analysis.

For ICP-MS analysis, about 500 mg of honey or crop samples, respectively, was weighed into glass tubes. 2 ml of HNO_3_ was added, and tubes were filled up with double distilled water to a final volume of 10 ml and vortexed to ensure sample homogeneity. Following this, microwave digestion was done with an Ultra Clave III (MLS Mikrowellen-Labor-Systeme GmbH, Leutkirch, Germany) where temperature was gradually increased from 80 to 200 °C at 900 W and 100 bar. Samples were cooled down and diluted adequately for ICP-MS analysis (NexION 300 X, PerkinElmer, Waltham, MA, USA). A ICP multi-element standard solution (Merck KGaA, Darmstadt, Germany) was used for calibration at concentrations of 0.1 µg/l, 0.2 µg/l, 1 µg/l, 10 µg/l, and 20 µg/l prepared with double distilled water and HNO_3_. A CertiPUR Rhodium ICP-standard solution (*c* = 1000 mg/l Rh, Merck KGaA, Darmstadt, Germany) served as internal standard (LOQ < 0.025 mg/kg).

### Statistics

The data were statistically analyzed using JMP Pro 16. Data on efficacy were checked with a Shapiro–Wilk test for normal distribution. Because of the non-parametric characteristics of the data, we used the Kruskal–Wallis test and a post hoc Dunn’s test including Bonferroni correction for multiple comparisons. For comparison of the 1st and 2nd brood assessments of each treatment we used the Wilcoxon Rank Sum test (Mann–Whitney test).

## Results

### Efficacy of different treatments

**Fig. 3:**
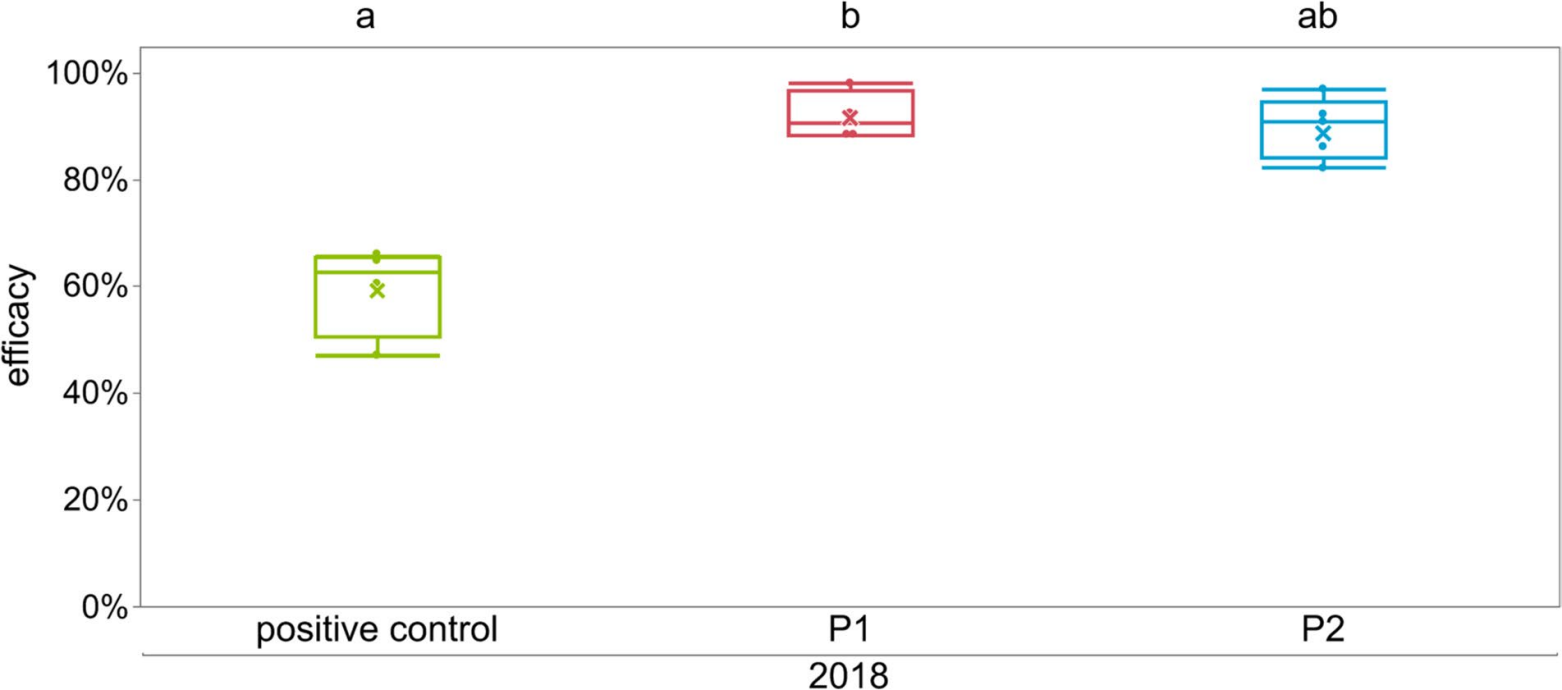
Efficacy of different treatments in the preliminary trials 2018 shown as box plots and mean indicated by the x. Positive control colonies were treated with 250 ml 60% formic acid (*n* = 4), P1 colonies were treated with 7 l of 25 mM LiCl syrup (*n* = 4), P2 with 4.5 kg 50 mM LiCl candy (*n* = 5). Significant differences are indicated by different letters (*p* < 0.05, Dunn’s test; additional data are given in Online Resource 3)

In the preliminary experiment in 2018, the broodless colonies treated with LiCl demonstrated an efficacy of 92.0 ± 4.5% for the syrup application (P1) where each colony received 7 l of 25 mM LiCl spiked Apiinvert®. For the candy application (P2), we applied 4.5 kg 50 mM LiCl which resulted in a mean food uptake of 2040 ± 786 g of candy per colony in 8 days (additional data are given in Online Resource 2) and an efficacy of 89.8 ± 5.6%, with no significant difference between the treatments (Fig. 3, *p* = 1, Dunn’s test, Online Resource 3). Treatment with LiCl outperformed the positive control treatment with formic acid, where only 59.5 ± 8.5% of the mites were killed. The formic acid treated colonies did not have caged queens, as the treatment penetrates into the brood nest, and we wanted to compare LiCl results with the most commonly used method of Varroa control in Germany.

**Fig. 4:**
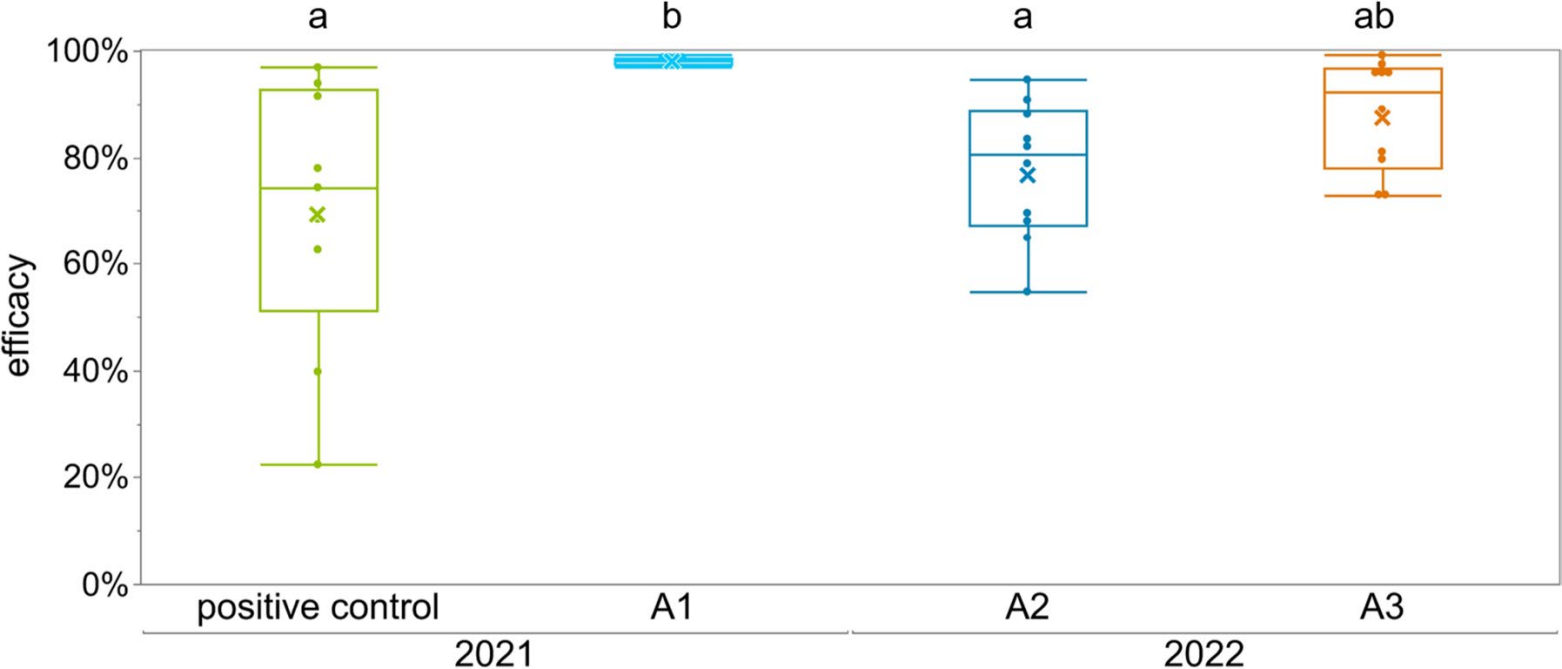
Efficacy of different LiCl administrations and positive control shown as box plots and mean by x. Positive control colonies (*n* = 9) were treated with 250 ml 60% formic acid. For A1 (*n* = 6) we applied 2.5 kg 50 mM LiCl candy over a period of 9 days; A2 colonies (*n* = 10) received 2 kg 50 mM LiCl candy in 5 days; for A3 (*n* = 10) we applied 0.5 kg of 50 mM LiCl candy four times in seven-day intervals (total of 2 kg candy) without caging the queen. Box plots with different letters are statistically different (Dunn’s test, *p* < 0.05, additional data are given in Online Resource 4)

In the years 2021 and 2022, LiCl was exclusively applied via candy (Fig. 4). For treatment A1 each colony received 2.5 kg 50 mM LiCl candy, which resulted in a mean food uptake of 1,739 ± 167 g in 9 days and the highest efficacy with 98.1 ± 0.7%, followed by A3 with 87.9 ± 10.5% and A2 with 77.5 ± 12.8%. For treatment A2 and A3 each colony consumed a total of 2 kg of 50 mM LiCl candy, which incorporates 3.1 g LiCl-salt (additional data are given in Online Resource 2). Positive control with formic acid was the least effective (69.8 ± 25.2%) and showed the highest variation between colonies, ranging in efficacy from 22.4% to 96.9%. Due to higher variances in A2 and A3, they did not differ significantly from the positive control (*p* = 1 and *p* = 0.6, respectively, Dunn’s test, Online Resource 4) (Fig. 4).

The total number of counted mites in 2021 showed a mean mite load per colony of 3,087 ± 915 for positive control colonies and 2,129 ± 967 for A1 colonies. Each colony had more than 1000 mites (Table 2). In 2022, the mean mite loads were on average 1429 ± 1155 mites for the A3 colonies and substantially lower in the A2 colonies with on average 456 ± 296 mites. Here, the lowest number of total counted mites per colony was 153 and 168, respectively.

**Table 2:** Total number of dead mites counted during the observation period (main treatment and follow-up treatment) for each treatment group are given together with the mean mites per colony ± standard deviation. Additionally, the range of mite counts per colony and the mean mites during follow-up treatment are given. Positive control colonies were treated with 250 ml 60% formic acid. For A1 we applied 2.5 kg 50 mM LiCl candy over period of 9 days; A2: 2 kg 50 mM LiCl candy in 5 days; A3: 4 × 0.5 kg 50 mM LiCl candy in seven-day intervals with no queen caging

Treatment	Year	Total mites	No. of colonies	Mean mites/colony	Range of mite counts/colony	Mean mites during follow-up treatment
Positive control	2021	27,783	9	3087 ± 915	1305–4710	933
A1	2021	12,776	6	2129 ± 96	1154–3508	36.3
A2	2022	4558	10	456 ± 296	168–1102	83.1
A3	2022	14,291	10	1429 ± 1155	153–3231	95.9

The mean number of mites killed per colony by the follow-up treatment was 933 in positive control colonies, 36.3 in colonies treated with A1, 83.1 in those treated with A2, and 95.9 for those treated with A3 (Table 2). This information can be used for assessing whether the LiCl treatments alone have reduced the mite population below the economic threshold.

### Honey bee brood survival

In total, we assessed 9463 brood cells from the freshly laid egg stage in 6 colonies of A1, 8 colonies of A2 and 10 colonies for both brood assessments of A3. Due to absence of eggs in two colonies on the assessment day, we could only examine the brood development for 8 of 10 colonies in A2.

**Fig. 5:**
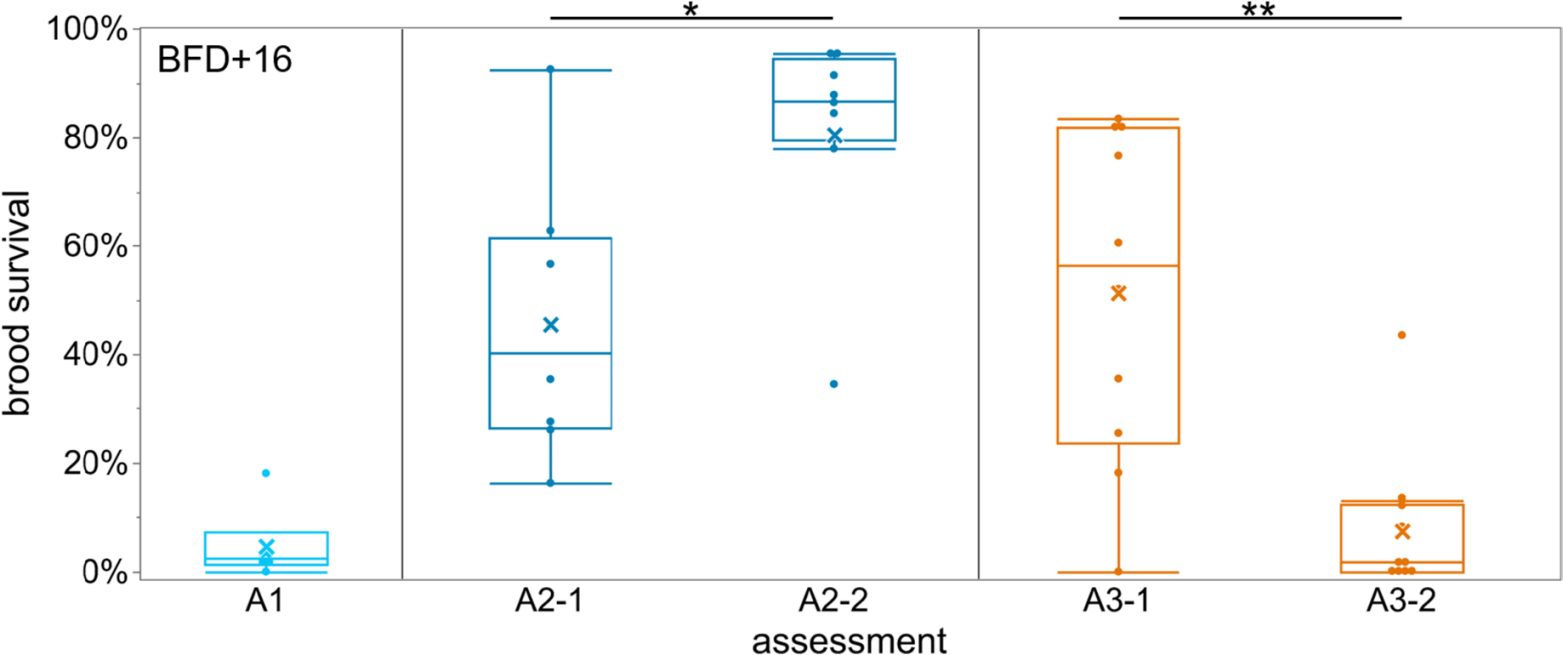
Brood survival rates in free flying colonies during different applications of 50 mM LiCl candy. A1: 2.5 kg of 50 mM LiCl candy over 9 days (*n* = 6); A2: 2 kg of 50 mM LiCl candy over 5 days (*n* = 8); A3: 4 x 0.5 kg 50 mM LiCl candy in seven-day intervals in colonies without queen caging (*n* = 10). For treatment A2 and A3, a subsequent brood assessment was carried out after completing the first one. Data given as boxplots for survival rate on day 16 post the brood fixing day (BFD), mean is given as an x. Total number of selected eggs on BFD were 982 (A1), 2574 (A2-1), 1600 (A2-2), 2049 (A3-1) and 2258 (A3-2). Significant differences between 1st and 2nd assessments are indicated with an asterisk (Wilcoxon Rank Sum test)

Treatment A1 with 9 days of LiCl treatment led to the highest brood removal rates and only 4.7% of the brood survived until BFD + 16 (Fig. 5). The first assessment of A2 (A2-1) showed a high variability in the survival probability of brood from the 8 treated colonies with a mean brood survival rate of 45%. The 2nd assessment (A2-2), which started 19 days after the LiCl application, had the highest brood survival rate with 82%, which was statistically different to the first assessment (*p* = 0.02, Wilcoxon Rank Sum test). The mean brood survival rate of the first assessment in A3 was 52% and thus similar to the first assessment of A2. However, in the 2nd brood assessment (A3-2), which started 2 days after the 3rd LiCl application, the brood survival rate decreased to only 8%, which was significantly lower than A3-1 (*p* = 0.004, Wilcoxon Rank Sum test).

#### Colony winter survival

In winter 2021, we lost one colony from the positive control group due to Nosema disease. The diagnosis was made through microscopic examination of sampled bees. In the 2022 experiments, we found one colony dead in the following spring probably due to queen loss in fall. In this colony, the queen was still present during the first brood assessment (A2-1); however, during the second assessment (A2-2), we were not able to find eggs. All the other colonies overwintered well.

### Distribution and detection of lithium

In colonies treated over a period of nine days (A1), we took 66 stored food and 61 adult bee samples over a period of 21 days. The 1st sampling was conducted before LiCl application, showing no lithium contamination in bees nor in food (Online Resource 5). Within five days of commencing the treatment, the concentration of lithium in the honey crop of bees sampled from the central area of the lower hive body rose to 69 mg/kg lithium and doubled four days later to reach the maximum of 131 mg/kg. After the remaining LiCl food was removed on day 9, the lithium concentration quickly dropped through day 17; thereafter it increased slightly during the last sampling 21 days post treatment. On no sampling day was there a significant difference between the lithium concentration from bees sampled from top or bottom hive body in our two brood chamber set-up. The increase in the lithium concentration in the honey crop was mirrored by the number of fallen mites. The mite drop reached a maximum on the fifth day, when a mean of 983 mites dropped, decreasing thereafter as shown in the figure (Online Resource 5).

In the stored food samples, we first detected lithium in uncapped cells on day 9 after commencing the LiCl treatment (25 mg/kg), whereas the first detection in capped cells was on day 13 (4.4 mg/kg). The lithium concentration was always lower in samples taken from capped cells and was significantly lower on day 17 with 5.2 mg/kg lithium compared to 36 mg/kg in open cells (*p* = 0.013, Tukey–Kramer HSD) (Online Resource 5).

**Fig. 6:**
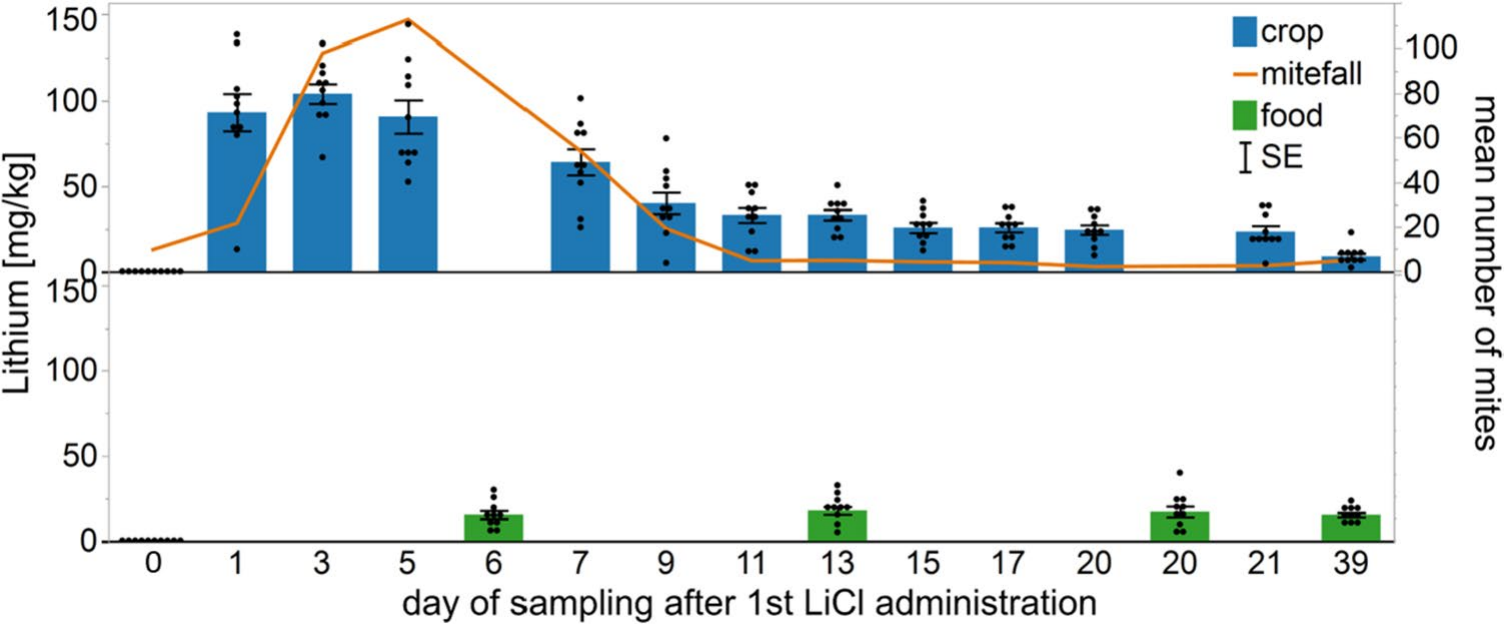
Distribution of lithium in the honey crop of sampled bees and in stored food from colonies (*n* = 10) during the five-day treatment (A2), each value given as the mean ± SE in the error bars. Administration of 2 kg of 50 mM LiCl candy started 1 day after the first sampling and was terminated five days later. The mean number of fallen mites for every two days is shown by the orange line. Follow-up treatment with Bayvarol® started on day 11

In 2022, when colonies were treated for five days (A2), 130 bee samples were taken from the treated colonies. From the colonies repeatedly treated for 4 × 2 days (A3) 170 bee samples were taken. Lithium concentration in the honey crop of bees from the A2 treatment displayed a similar distribution as in A1 group with a peak of 104 mg/kg lithium on day 3, decreasing after termination of treatment (Fig. 6). Maximum lithium concentration in stored food occurred 13 days after the start of the treatment (18 mg/kg).

**Fig. 7:**
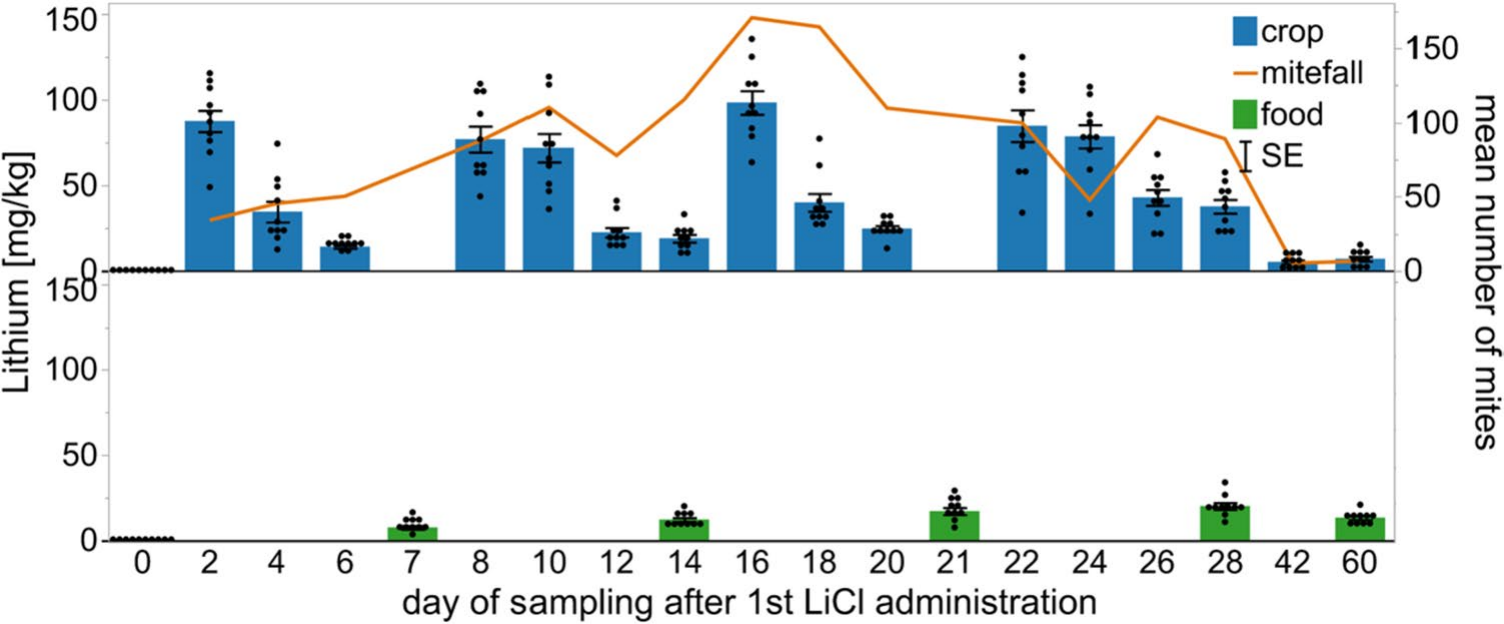
Distribution of lithium in the honey crop of sampled bees and stored food from colonies (*n* = 10) during the repeated short-term treatment (A3), each value given as the mean ± SE in the error bars. Administration of each 0.5 kg 50 mM LiCl candy took place on days 0, 7, 14, and 21. The mean number of fallen mites for every 2 days is shown as the orange line. Follow-up treatment with Bayvarol® started on day 30

For the repeated short-term treatments used in A3, we detected several short peaks after each application of the 0.5 kg LiCl candy (Fig. 7). The highest concentration in the honey crop was found on day 16 after the 3rd application with 98 mg/kg lithium. The concentration of lithium in the sampled food reached a maximum on day 28 with 20 mg/kg. For both treatments, A2 and A3, we took the last samples 39 days after the last application of LiCl and measured 15.4 mg/kg lithium in the A2-treated colonies and 13.2 mg/kg in the A3-treated colonies.

The stored food sampled from brood chambers in Spring 2023 revealed low concentrations of 5.4 ± 2.4 mg/kg lithium in A2-treated colonies and an even lower concentration of 3.1 ± 2.4 mg/kg in the A3 treatment group (Table 3). In the freshly collected nectar from honey chambers, we measured 0.8 ± 0.7 mg/kg lithium from A2 colonies, which was further reduced to 0.1 mg/kg in the harvested honey. For A3, we measured 0.2 ± 0.2 mg/kg and 0.2 ± 0.2 mg/kg, respectively (Table 3).

**Table 3:** Lithium concentration in spring 2023 of samples from stored food of brood chambers, freshly collected nectar in honey chambers and harvested honey from colonies treated in summer 2022. Each given as mean ± standard deviation for the treatment groups. Numbers of analyzed samples are given in brackets

	Lithium [mg/kg]		
Treatment method	Brood chamber	Honey chamber	Honey
A2	5.4 ± 2.4 (*n* = 9)	0.8 ± 0.7 (*n* = 6)	0.1 ± 0 (*n* = 4)
A3	3.1 ± 2.4 (*n* = 10)	0.2 ± 0.2 (*n* = 5)	0.2 ± 0.2 (*n* = 4)

## Discussion

Our field experiment demonstrated high efficacy of LiCl in colonies with and without brood when applied by feeding in syrup or candy. In the preliminary experiments in the year 2018 with broodless colonies, we already reached a mean efficacy of more than 90% with both the syrup and the candy application. We then decided to continue with the candy treatment, because of the slower consumption by the bees which enables a continuous flow of LiCl into the colony for more than one week through a single feeding. The field test with broodless colonies in the year 2021 revealed an even higher efficacy of 98%, which was significantly different from colonies treated with the standard formic acid Varroa control in the same apiary. This confirmed that the high efficacy of LiCl, which had previously been demonstrated by Kolics et al. (2021b) with the trickling method, can also be achieved using a single application of 2.5 kg candy spiked with 50 mM LiCl. The next field test with broodless colonies in the year 2022 had only a 78% efficacy. Two reasons could potentially have caused this significantly lower efficacy compared to the 2021 study. First, we reduced the duration of the treatment from 9 to 5 days to reduce the impact on brood loss in the first subsequent brood cycle seen in the 2021 experiment (see below). But this shortened period was obviously not sufficient to achieve a high efficacy. Second, the mean mite load per colony was 456 mites compared to 2129 in the year before and some colonies had less than 200 mites. In colonies with relatively low mite numbers, the reinvasion of mites in late summer and early fall (Frey and Rosenkranz 2014), a common occurrence in apiaries in this part of Germany, can have a greater impact on the calculation of the efficacy. Our experimental apiaries were not in the direct vicinity of colonies managed by other beekeepers, however even over a distance of 1.5 km an invasion of more than 100 mites per colony is possible (Frey et al. 2011).

Our repeated short-term LiCl treatments of colonies with brood revealed an efficacy of 88% and confirmed the results of former field tests with lithium salts in brood rearing colonies (Stanimirovic et al. 2022), however at the cost of high brood removal rates (see below). The efficacy of a treatment is not the only important value, rather it is crucial whether the LiCl treatments reduce the absolute number of mites in all colonies below the economic threshold. This economic threshold can be estimated in various ways such as the natural daily mite drop or the infestation in sampled adult bees. Jack and Ellis (2021) defined an infestation rate of the adult bees of 2–3% as a general accepted economic threshold. In our follow-up treatments directly after the LiCl applications we killed an average of only 36–96 mites per colony. With an average number of about 15,000–20,000 bees in our colonies at the end of September, this number of mites represents an infestation of considerably less than 1%. Therefore, we consider LiCl applications in late summer as sufficiently effective to reduce the Varroa infestation below the economic threshold, providing beekeepers with a highly effective alternative treatment option once it is approved for use.

Our analysis of honey bee crops and stored food for traces of lithium demonstrated an even distribution of the active ingredient lithium within the colony, despite food uptake differing somewhat between colonies. This low variance between colonies in the distribution of the active ingredient lithium and the efficacy of treatment results in high levels of treatment success throughout an apiary, which cuts down on varroa reinvasion and potential transfer of mites between strong and weak colonies (Giacobino et al. 2023).

In contrast to currently available varroacides, our application method of LiCl takes advantage of the social behavior of food sharing in bees, called trophallaxis (LeBoeuf 2017). This leads to a rapid distribution of the treatment food throughout the whole colony (Crailsheim 1998; Nixon and Ribbands 1952) with sufficient amounts of lithium to kill Varroa mites in sampled bees within 48 h of application, as demonstrated by the spike in the mite drop within two days of LiCl administration (see Fig. 6). We always detected substantially lower levels of lithium in the food samples than in the crop of the sampled bees. In 2021, when about 1.7 kg of a 50 mM LiCl candy was applied per colony, the maximum lithium content found in open food cells was 36 mg/kg, whereas in the crop it rose to 131 mg/kg. In 2022, when the feeding period was reduced from 9 to 5 days, the maximum lithium levels were somewhat lower, with similarly low levels in food compared to bee crops as seen in 2021. To produce honey from nectar or from a beekeeper applied food source like syrup or candy, honey bees rework the food source multiple times, before permanently storing it in a cell (Park 1925). During this food processing, the bee’s ventriculus removes contaminations and excess water. Honey bees also have the capacity to remove heavy metals from collected nectar in the process of producing honey (Borsuk et al. 2021), which could help explain why we always detected substantially lower lithium concentrations in stored food compared to crop samples.

As shown in previous studies, exposure to LiCl during larval development can result in high brood mortality (Rein et al. 2022). We thus paid particular attention to the survival of the first batch of brood reared after release of the queen. In 2021, the long treatment period of 9 days resulted in low brood survival, most likely due to an overlap of applied LiCl food and the presence of the first larvae. To avoid this loss of brood, we shortened the treatment period to 5 days in 2022, reducing the exposure of the sensitive early larval stages to LiCl. Brood survival increased, but with high variance in survival rates among the colonies. We assessed a second brood cycle, which showed that there are no long-term effects to brood survival from LiCl as was also observed in 2021. This is also supported by the fact that 34 colonies treated with LiCl (from all experiments) overwintered well and only one colony died due to queen loss in fall 2022. Thus, although we see a short time frame of brood loss immediately after treatment, the colony then successfully rears brood and does not suffer any population loss from this brief interruption.

Other studies tested different LiCl applications in broodless colonies with even higher concentrations, but failed to evaluate the aftereffects on brood reared post treatment (Kolics et al. 2022). Stanimirovic et al. (2022) carried out lithium citrate treatments in colonies with brood and stated that there were no side effects observed during the year, however the authors did not analyze potential carryover effects of lithium citrate on brood directly after the treatment applications. Our experiments with repeated short-term treatments in colonies with a free-roaming queen and brood (A3) clearly confirm the low tolerability of honey bee larvae for LiCl. The goal with this new application method was to limit the contact of larvae to LiCl for a maximum of 1–2 days by feeding the treatment repeatedly in small amounts. However, this did not provide the desired reduction in brood loss. Both the first and second brood assessment during application resulted in low survival rates and so clearly a treatment with LiCl in colonies with brood is only possible at the cost of lost brood during the application period. As seen in our studies, the side effects on brood only occur during and shortly after the period of the treatment when lithium contaminated food is still circulating among the nurse bees.

From our comprehensive studies on LiCl, we can conclude the following regarding efficacy, distribution of the compound within the colony and side effects on brood for the treatment of broodless colonies:

1. A single feeding of 2 kg of 50 mM LiCl candy kills more than 95% of Varroa mites under field conditions when exposure lasts 9 days. It could potentially be shortened, but 5 days was too short to achieve the required 90% efficacy for Varroa mite control.
2. Side effects on the honey bee brood occurred only in the first brood cycle laid directly after the end of the application. If queens were released one week after treatment cessation, these side effects could largely be prevented.
3. Our data on the concentration and distribution of lithium within the worker bees of a treated colony indicate that within 48 h lithium levels reach their maximum level within the bee’s crop and remain elevated for the duration of the treatment. Even some days after the end of the application the lithium concentrations within the crops of the bees should still be high enough to kill the parasitizing mites.

These results suggest that a promising strategy for future applications would be to start the treatment via candy one week before the release of the queen, when the colony only contains capped brood. The lithium concentration in the bees at the time of queen release should be sufficient to kill the few mites emerging with the last brood cells, yet subside to a harmless level by the time the first larvae hatch from queen laid eggs.

The long-term circulation of lithium in colonies post treatment and thus the potential risk of residue accumulation in the honey must be investigated before lithium-salts can be authorized as a varroacide. Lithium occurs naturally in some honeys (up to 15.6 mg/kg) (Abdulkhaliq and Swaileh 2017; Bogdanov et al. 2008; Conti et al. 2018; Tariba Lovaković et al. 2018), mineral water (1.7–1725 µg/l) (Seidel et al. 2019), and even other beverages like wine and soft drinks (Seidel et al. 2020). We even found lithium at a concentration of 0.27 mg/kg in the Apiinvert® syrup sold as bee feed on the German market. It is thus hard to define an acceptable residue level, though the naturally occurring range in honey suggests a higher limit than with other varroacide residues is warranted. What is considered acceptable is currently debated; Kolics et al. (2021a) found an increase in lithium in uncapped honey directly after application but claimed a “full recovery” by day 22 with a concentration below 0.25 mg/kg. Yet they found a concentration of 22.4 mg/kg in the ripe honey on day 28. In contrast, Stanimirovic et al. (2022) found a significant difference of lithium in honey taken from honey chambers of untreated colonies (0.018 mg/kg) compared to 0.034 mg/kg from lithium treated colonies, which is still far below the naturally occurring concentrations of lithium in honey.

Another study detected lithium concentrations seven days post-treatment between 0.05 and 0.9 mg/kg (Prešern et al. 2020). Our residue analysis of honey and stored bee food was conducted in the spring of the following year, which was almost ten months post treatment. We found lithium at rates of 3.1 to 5.4 mg/kg in the stored food of the brood chamber. Colonies were fed an additional 15–20 kg of sugar syrup post treatment to provide them with enough winter food stores, which must have diluted the lithium concentrations. In freshly made spring honey we harvested, we only found 0.1–0.2 mg/kg lithium which is far below the natural occurring concentrations. Our treatment method of feeding LiCl in candy does not lead to undesirable residues in honey.

LiCl provides many advantages compared to current varroacides on the market. The most frequently used non-synthetic varroacides in Middle and Northern Europe are currently formic acid in the summer and oxalic acid in the winter, both of which require specific environmental or colony conditions for high efficacy and good tolerability (Adjlane et al. 2016; Steube et al. 2021). Our new application method, unlike these organic acids, allows the beekeeper to be independent of environmental factors. Even though caging the queen, in order to create a broodless period requires additional effort and time, especially in large beekeeping operations like in the USA or Canada, such caging is becoming more standard as a method of integrated Varroa control (Büchler et al. 2020; Gregorc et al. 2017; van der Steen and Vejsnæs 2021). A brood interruption not only has the advantage that all mites are forced to reside on the adult bees, which makes them vulnerable to varroacides, it also interrupts the mite’s reproductive cycle which slows down this parasite’s population growth (Gabel et al. 2023; Jack and Ellis 2021; Rosenkranz et al. 2010). In the colonies with the repeated treatments where we allowed bees to keep rearing brood, three times as many mites fell (compare A3 with brood to A2 without brood in Table 2), even though the natural mite drop before the treatment was similar in both groups. Giacomelli et al. (2016) showed that queen caging alone resulted in a 40% reduction of Varroa populations, but should always be used in combination with a varroacide to achieve sufficient mite mortalities. As LiCl is a natural salt, the combination of queen caging and LiCl application could be a treatment option for organic beekeepers, too. A comparison of the additional workload generated by different summer brood interruption methods showed that queen caging was one of the least labor-intensive ones with no negative impact on colony strength between the different methods (Büchler et al. 2020). Rather, Lodesani et al. (2014) demonstrated that in Italy treatments that include a brood interruption approach yielded the greatest colony survival rate compared to alternative treatment methods. A broodless period during late summer allows beekeepers to remove old combs and replace them with new ones, reducing pesticide residues in the lipophilic beeswax, which has been shown to have a beneficial effect on the productivity of the colony (Berry and Delaplane 2001; Taha et al. 2021).

## Conclusion

When caging the queen is integrated into a varroa treatment strategy, the application of LiCl via candy feeding represents an effective and “easy to apply” treatment due to its rapid action and even distribution within a colony, regardless of external weather conditions. The systemic mode of action makes LiCl an attractive new varroacide that deserves more attention and a communal effort to bring this new Varroa control tool to the market, where it can aid beekeepers in treating infested colonies sustainably and keeping their bees healthy.

### Supplementary Information

Below is the link to the electronic supplementary material.

Supplementary file1 (PDF 350 KB)

## Data Availability

The datasets generated during and analyzed during the current study are available from the corresponding author on reasonable request.
