# Supplementary material for: Lithium chloride treatments in free flying honey bee colonies: efficacy, brood survival, and within-colony distribution
Source: Parasitol Res. 2023 Dec 22;123(1):67. doi: 10.1007/s00436-023-08084-y (PMC10746590; doi:10.1007/s00436-023-08084-y)

Supplemental Information

Lithium chloride treatments in free flying honey bee colonies: efficacy, brood survival, and within-colony distribution

Journal of Parasitology Research

Carolin REIN[1], Marius BLUMENSCHEIN[1], Kirsten TRAYNOR[1], Peter ROSENKRANZ[1]

[1]State Institute of Bee Research, University of Hohenheim, D-70599 Stuttgart, Germany

Corresponding author: C. Rein, carolin.rein@uni-hohenheim.de; ORCID: 0000-0003-2764-3025

1: Sampling protocol for 2021. The 2nd column shows the bee sampling; the 3rd column indicates the sampling of the stored food. Bees were sampled in four-day intervals from a central frame from top and bottom box of six colonies. Food samples were taken from capped and uncapped cells on the same day. Treatment indicates the application of the 50 mM LiCl candy

Day	Bee sampling 2021	Food sampling 2021
0	central, top	uncapped food
1 treatment		
2 treatment		
3 treatment		
4 treatment		
5 treatment	central, top + bottom	uncapped + capped food
6 treatment		
7 treatment		
8 treatment		
9 treatment	central, top + bottom	uncapped + capped food
10 post- treatment		
11 post- treatment		
12 post- treatment		
13 post- treatment	central, top + bottom	uncapped + capped food
14 post- treatment		
15 post- treatment		
16 post- treatment		
17 post- treatment	central, top + bottom	uncapped + capped food
18 post- treatment		
19 post- treatment		
20 post- treatment		
21 post- treatment	central, top + bottom	uncapped + capped food

2: Food consumption of 50 mM LiCl candy for each colony, along with the mean consumption in 2018 and 2021, as well as the total amount of LiCl-salt consumed. In 2022, all 10 colonies in both treatment groups consumed 2,000 g candy either in a single or via 4 repeated treatments of 500 g candy

**Year**	**Food uptake per colony [g]**						**Mean ± SD**	**Used LiCl per mean [g]**
**2018**	1,200	3,000	2,900	1,200	1,900		2,040±786	3.2
**2021**	1,833	1,648	1,877	1,547	1,980	1,550	1,739±167	2.7
**2022**	2,000							3.1

3: Statistics of efficacy in preliminary trials 2018. Kruskal-Wallis test and post hoc Dunn's test

**Category 1**	**Category 2**	**Score Mean Difference**	**Standard Error Difference**	**Z**	**p-Value**
P2	Positive control	7.000	2.754	2.542	0.033*
P1	Positive control	5.675	2.612	2.172	0.09
P2	P1	-1.125	2.612	-0.431	1.0

4: Statistics of efficacy in main experiment 2021, 2022. Kruskal-Wallis test and post hoc Dunn's test

**Category 1**	**Category 2**	**Score Mean Difference**	**Standard Error Difference**	**Z**	**p-Value**
A1	Positive control	19.472	5.400	3.606	0.002*
A3	A2	8.506	4.708	1.807	0.425
A3	Positive control	7.500	4.582	1.637	0.610
A2	Positive control	0.906	4.708	0.192	1.0
A3	A1	-10.867	5.291	-2.054	0.240
A2	A1	-18.467	5.291	-3.490	0.003*

5: Distribution of lithium during 9 day 50mM LiCl candy administration (A1, 2021) in crop of sampled bees from upper and lower frames, and food of open and capped cells, each given as mean ± SE (n=6). Also the mean number of fallen mites is shown as orange line. No significant difference between crop samples from upper and lower frame on any day. Only food samples on day 17 show significant difference p=0.013 (Unifactorial ANOVA followed by Tukey-Kramer HSD)

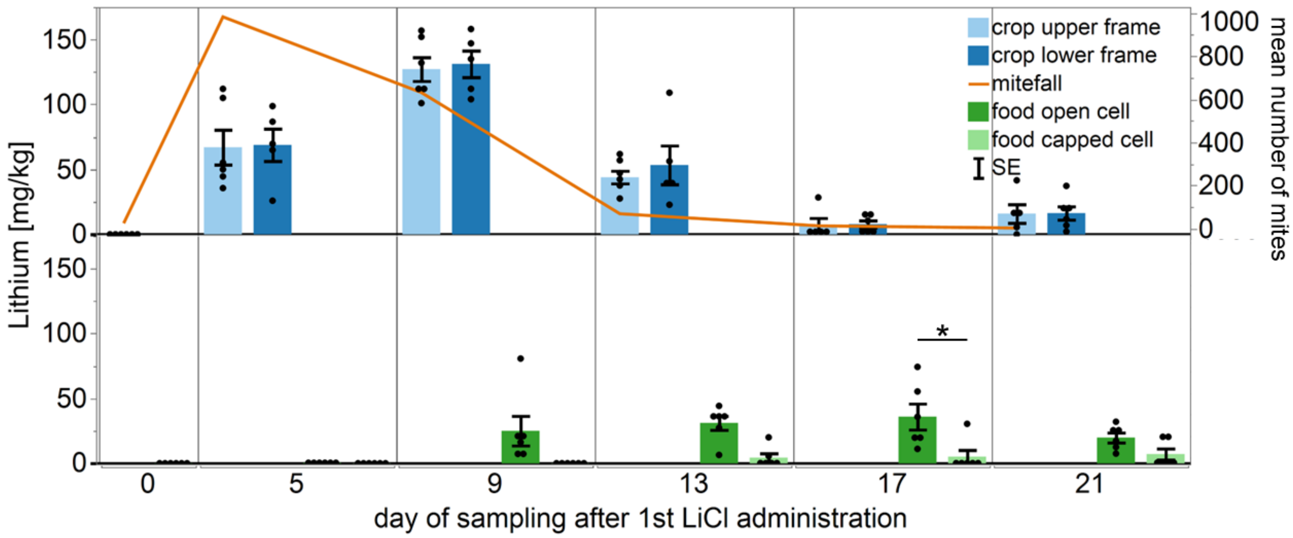

Supplement: Supplementary file 1 — Supplementary file1 (PDF 350 KB) [file 436_2023_8084_MOESM1_ESM.pdf]
